# Increasing consensus on terminology of Achilles tendon-related disorders

**DOI:** 10.1007/s00167-021-06566-z

**Published:** 2021-05-15

**Authors:** K. T. M. Opdam, R. Zwiers, J. I. Wiegerinck, C. N. van Dijk, Ankleplatform Study Collaborative –Science of Variation Group, C. Topliss, A. R. Gaspar, N. Moreno, A. Puttaraju, Y. J. Lau, A. Grauls, C. Nery, A. D. Mora, D. Tsoukas, I. Spanos, N. Koukoulias, F. Lijoi, Y. Yasui, D. Guzenko, M. van Sterkenburg, P. Zbikowski, B. Sadlik, M. Santos Carvalho, P. Rasovic, V. Stevanovic, C. Blasco, N. Atallah Yordi, J. Batista, C. Lucca Stoffel, N. Gomes Júnior, F. Martinez, J. M. Leblanc, G. Pánics, S. Varghese, S. Rajagopalan, A. Gavaskar, A. Marmotti, V. Kimtys, G. Hajduk, P. Ferrao, P. Hemmingsson, M. Golovakha, O. Lahoti, J. Davenport, J. Mcwilliam, G. van Gompel, F. Krappel, Y. Zhu, G. Antoniades, F. Flores Santos, S. G. Batibay, S. Verfaillie, J. Brandão, A. L. Rocha de Souza, M. Vuldzhev, D. Xiang, O. Castro Aragon, I. Bojanic, I. Rakovac, H. Haapasalo, A. Toom, C. Plaaß, M. Baacke, H. Waizy, N. Dreiangel, E. Palmanovich, N. Martinelli, A. Ortolani, P. Sicchiero, J. Sasahara, L. Gomez-Carlin, G. Kerkhoffs, C. van Bergen, G. Bulstra, M. Andersen, A. Wojciech, A. Boszczyk, G. Martinho, J. Vide, M. Sousa, E. Sorokin, J. Lansdaal, S. Al-Nammari, T. Syed, V. Upadhyay, I. Bissell, M. Dunning, A. Ajis, B. Rudge, M. Pinheiro, J. del Vecchio, R. Freihaut, C. Brown, M. Van den Bogaert, M. Cesar Mattos e Dinato, M. Viana Pereira Filho, C. Bustamante, J. Kalb, D. Nikolopoulos, D. Hatziemmanuil, P. Symeonidis, T. Vasilakakos, T. Thorvardarson, J. Walsh, G. Favilli, P. Guidi, S. Abdulsalam, P. Spennacchio, M. van den Bekerom, A. Bertz, H. Liszka, H. Pereira, A. Ramos, R. Marinescu, J. Azevedo, A. Engvall, G. Cserhati, B. Sghaier, O. Aiyenuro, C. Marquis, T. Barwick, C. Gross, E. Pereira, V. Pasters, M. Monteagudo, M. Orduña-Moncusí, S. Burtt, S. Chandrashekar, H. Shalaby, R. Thomas, H. Kurup

**Affiliations:** 1 Department of Orthopedic Surgery, Amsterdam UMC, PO Box 22660, 1100 DD Amsterdam, The Netherlands; 2 Academic Center for Evidence Based Sports Medicine (ACES), Amsterdam, The Netherlands; 3 Amsterdam Collaboration for Health and Safety in Sports (ACHSS), Amsterdam, The Netherlands; 4 Department of Orthopedic Surgery, Bergman Clinics Rijswijk, Rijswijk, The Netherlands; 5 FIFA Medical Centre of Excellence Ripoll-dePrado-vanDijk SportClinic Madrid, Madrid, Spain; 6 FIFA Medical Centre of Excellence Clinica do Dragao Porto, Porto, Portugal

**Keywords:** Terminology, Achilles tendon, Mid-portion Achilles tendinopathy, Insertional Achilles tendinopathy, Retrocalcaneal bursitis

## Abstract

**Purpose:**

Aims of this study are to evaluate the current terminology and assess the influence of the latest proposals on the terminology used for Achilles tendon-related disorders in both daily practice and literature.

**Methods:**

(1) All orthopedic surgeons experienced in the field of foot and ankle surgery of the Ankleplatform Study Group were invited to participate in this survey by email. They were requested to fill out a survey on terminology in six typical cases with Achilles tendon-related disorders. (2) A systematic literature search of Achilles tendon-related disorders was performed in eight foot and ankle journals in Medline, Embase (Classic) from 2000 to 2016. All extracted terms were counted and compared to the terminology proposals, based on anatomic location, symptoms, clinical findings and histopathology.

**Results:**

(1) In total, 141 of the 283 (50%) orthopedic surgeons responded to the survey. In five out of six cases with Achilles tendon-related disorders, the majority gave an answer according to latest proposals. (2) An overview of terminology used for Achilles tendon-related disorders from 2000 to 2016 shows an increase in use of terminology according to the latest proposals based on anatomic location, symptoms, clinical findings and histopathology.

**Conclusion:**

The revised terminology for Achilles tendon-related disorders based on anatomic location, symptoms, clinical findings and histopathology is used by the majority of orthopedic surgeons and is increasingly used in the literature. However, the indistinct Haglund eponyms are still frequently used in Achilles tendon-related terminology.

**Level of evidence:**

Level IV.

**Supplementary Information:**

The online version contains supplementary material available at 10.1007/s00167-021-06566-z.

## Introduction

Several Achilles tendon-related disorders can be distinguished and for each pathology different definitions and terms or eponyms arose over time. As a result, the terminology for Achilles tendon-related disorders is inconsistent and confusing [8, 17].

Initially terms were used such as “cellulite peritendineuse”, “tendinitis Achillae traumatica”, “paratendinitis”, “tenosynovitis” and “peritendinitis” [10, 18]. The term ‘achillodynia’ was introduced as a descriptive term for Achilles tendon-related pain [1]. Subsequently terms were based on histological findings and a subdivision was made into insertional and non-insertional Achilles tendon problems [7, 14, 16]. Maffulli et al. [12] observed that terminology used for tendon conditions was misused and confusing. In their opinion definitions as tendinitis, tendinosis and paratendonitis can only be diagnosed after biopsy; however, they were often used in clinical practice without histopathologic examination. Due to a lack of consistence in nomenclature, Maffulli et al. advocated to use the term tendinopathy to describe clinical overuse conditions around the tendon characterized by pain, swelling and impaired performance [12]. Depending on the affected tissue, the terms tendinopathy, paratendinopathy or pantendinopathy were proposed.

In 2011, an addition was proposed to further purify the terminology used in Achilles tendon-related disorders to effectuate uniform and clear terminology [24]. This terminology is based on anatomic location, symptoms, clinical findings and histopathology and consists of the following five terms: mid-portion Achilles tendinopathy, insertional Achilles tendinopathy, Achilles paratendinopathy, retrocalcaneal bursitis and superficial calcaneal bursitis [24].

Uniform terminology provides the ability to communicate with an universal language in daily practice amongst clinicians and researchers. The aims of this study are to evaluate the current terminology and assess the influence of the latest proposals on the current terminology used for Achilles tendon-related disorders in both daily practice and literature.

## Materials and methods

This study consists of two parts, a survey amongst orthopedic surgeons on terminology in six typical cases with Achilles tendon-related disorder and a systematic search of the literature.

### Survey

Members of the Ankleplatform Study Group—Science of Variation Collaborative were invited. All orthopedic surgeons, experienced in the field of foot and ankle surgery, were invited by mail to log on to the website—www.ankleplatform.com— and were requested to fill out their demographics characteristics and a questionnaire.

Six typical cases with Achilles tendon-related disorders were presented (see Appendix I) [24]. Participants were asked to give their preferred diagnosis for each case presented. A reminder was sent after 2 weeks. Incomplete questionnaires were excluded from the study.

### Literature search

All terms described in publication about terminology of Achilles tendon-related disorders in 2011 were used [24]. Literature was reviewed for the terminology used in papers on Achilles tendon-related disorders and thereafter a systematic literature search was performed (see Appendix II for search strategy).

Eight journals in the field of foot and ankle surgery were selected: the American Journal of Sports Medicine, British Journal of Sports Medicine, Knee Surgery Sports Traumatology Arthroscopy, Foot & Ankle International, Journal of Orthopaedic Research, Acta Orthopaedica, Journal of Foot and Ankle Research and Journal of Foot and Ankle Surgery. All articles on Achilles tendon-related disorders, except Achilles tendon ruptures, published from 2000 until 2016 were included. Title and abstract were screened and the used terminology was extracted. All extracted terms were counted and divided into “according to the latest proposals” and “not according to the latest proposals”, based on anatomic location, symptoms, clinical findings and histopathology, which was published January 2011 (see Table 1) [24]. When multiple terms were used in one publication, for example mid-portion Achilles tendinopathy and insertional Achilles tendinitis, this was scored as “not according to the latest proposals”.

**Table 1:** The latest proposed terminology by van Dijk et al. [24]

Mid-portion Achilles tendinopathy	A clinical syndrome characterized by a combination of pain, swelling and impaired performance. It includes but is not limited to, the histopathological diagnosis of tendinosis
Insertional Achilles tendinopathy	This is located at the insertion of the Achilles tendon onto the calcaneus, bone spurs and calcifications in the tendon proper at the insertion site may exist
Achilles paratendinopathy	An acute or chronic inflammation and/or degeneration of the thin membrane around the Achilles tendon. There are clear distinctions between acute paratendinopathy and chronic paratendinopathy, both in symptoms as in histopathology
Retrocalcaneal bursitis	Is an inflammation of the bursa in the recess between the anterior inferior side of the Achilles tendon and the posterosuperior aspect of the calcaneus (retrocalcaneal recess)
Superficial calcaneal bursitis	Inflammation of the bursa located between a calcaneal prominence or the Achilles tendon and the skin

### Statistical analysis

All collected data were imported into Statistical Package for Social Sciences (SPSS) version 25.0 (SPSS Inc. Chicago, IL). Analyses of outcome data was descriptive. Continuous outcome measures were presented as mean with standard deviation for data with a normal distribution and as median with interquartile range in case of non-normal distributed data. Distribution of continuous variables was assessed using the Kolmogorov–Smirnov test. Descriptive data were presented as frequencies with percentages in case of categorical data.

## Results

### Survey

In total, 283 orthopedic surgeons were invited by mail of which 141 participated in the study (response rate 50%). Respondents originated from 50 different countries, the most common country of origin was United Kingdom (7%), followed by Portugal (3%), the Netherlands (3%), and Italy (3%). Thirteen participants did not complete the questionnaire and were, therefore, excluded. Table 2 shows the demographic characteristics.

**Table 2:** Demographics

	*N* = 141 (100%)
Male	133 (94.3)
Female	8 (5.7%)
Age	Median 40.0 (IQR 37.5–46)
Years in practice	Median 10 (IQR 6–16)
Number of patients with Achilles pathology each year	Median 50 (IQR 30–100)

Table 3 presents the preferred diagnosis in each of the six cases. Only in case 5, the majority gave a diagnosis not according to the latest terminology proposals, namely Haglund’s disease instead of retrocalcaneal bursitis.

**Table 3:** Overview of case answers

Case	1	2	3	4	5	6
	*N* (%)	*N* (%)	*N* (%)	*N* (%)	*N* (%)	*N* (%)
	128 (100)	128 (100)	128 (100)	128 (100)	128 (100)	127 (100)
Achilles pantendinopathy	–	–	7 (5.5)	–	–	7 (5.5)
Achilles tendinitis	4 (3.1)	–	13 (10.2)	–	–	2 (1.6)
Achilles tendinopathy	18 (14.1)	–	1 (0.8)	1 (0.8)	–	10 (7.9)
Achilles tendinosis	19 (14.8)	–	2 (1.6)	–	–	6 (4.7)
Achilles tendon bursitis	–	9 (7.0)	–	–	–	1 (0.8)
Achillodynia	1 (0.8%)	–	–	–	–	–
Achillotendinitis ossificans	–	–	–	6 (4.7)	–	–
Acute Achilles paratendinopathy	–	–	62 (48.4)	–	–	3 (2.4)
Bursitis Achillea	–	7 (5.5)	1 (0.8)	–	–	–
Cellulite peritendineuse of the Achilles tendon	–	–	6 (4.7)	–	–	1 (0.8)
Chronic Achilles paratendinopathy	4 (3.1)	–	4 (3.1)	–	–	46 (36.2)
Haglund’s deformity	–	6 (4.7)	–	2 (1.6)	24 (18.8)	–
Haglund’s disease	–	4 (3.1)	–	5 (3.9)	31 (24.2)	–
Haglund's exostosis: pump-bump, calcaneus altus, high prow heels, knobbly heels, cucumber heel	–	13 (10.2)	–	10 (7.8)	11 (8.6)	–
Haglund’s syndrome	–	1 (0.8)	–	1 (0.8)	23 (18.0)	–
Insertional Achilles tendinopathy	–	16 (12.5)	–	86 (67.2)	1 (0.8)	–
Mid-portion Achilles tendinopathy	39 (30.5)	–	–	1 (0.8)	–	14 (11.0)
Paratendinitis	1 (0.8)	–	11 (8.6)	–	–	4 (3.1)
Peritendinitis	–	–	10 (7.8)	–	–	2 (1.6)
Retrocalcaneal bursitis	–	17 (13.3)	1 (0.8)	1 (0.8)	29 (22.7)	–
Superficial Calcaneal bursitis	–	42 (32.8)	–	–	–	–
Tendinitis Achillea traumatica	–	–	–	–	–	–
Tenosynovitis	–	–	6 (4.7)	–	–	1 (0.8)
Midportion Achilles tendinopathy and paratendinopathy combined	34 (26.6)	1 (0.8)	1 (0.8)	1 (0.8)	–	28 (22.0)
Insertional Achilles tendinopathy and retrocalcaneal bursitis combined	–	10 (7.8)	–	10 (7.8)	6 (4.7)	–
Other	8 (6.3)	1 (0.8)	3 (2.3)	5 (3.9)	3 (2.3)	2 (1.6)

### Literature review

**Fig. 1:**
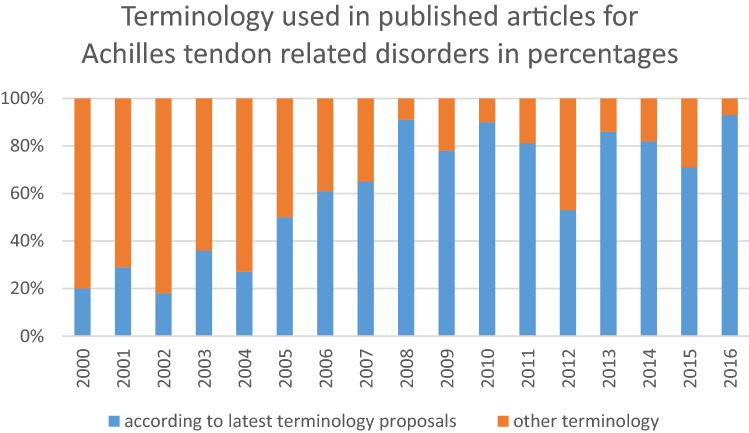
Overview of terminology used for Achilles tendon-related disorders in percentages of published articles (Y-axis) over the years (X-axis)

After the search, 257 articles remained for review. Thirteen articles were excluded based on other pathology than Achilles tendon pathology and 244 articles remained. Table 4 presents an overview of the numbers of times the terms were used in literature from 2000 to 2016. The most used terms are (chronic) Achilles tendinopathy, mid-portion Achilles tendinopathy and (chronic) Achilles tendinosis. Also, eponyms are still frequently used. Figure 1 provides an overview of the distribution of terminology used for Achilles tendon-related disorders according to the latest proposal and terminology not according to the latest proposals in percentages from 2000 to 2016. In 2000, 20% used terminology according to the latest proposals based on anatomic location, symptoms, clinical findings and histopathology and in 2016, 93%.

**Table 4:** Overview of the used terms in literature from 2000 to 2016

Number of times	Term
70	(chronic) Achilles tendinopathy
45	Mid-portion Achilles tendinopathy
29	(chronic) Achilles tendinosis
23	Insertional Achilles tendinopathy
11	(chronic) non-insertional Achilles tendinopathy
11	insertional Achilles tendinosis
9	Achilles tendinitis
8	Mid-portion Achilles tendinosis
7	Haglund’s deformity
6	Retrocalcaneal bursitis
5	Haglund’s syndrome
4	mid-substance Achilles tendinopathy
2	Haglund’s disease
2	insertional tendinitis
2	Achillodynia
1	Achilles paratendinitis, Achilles paratendinopathy, Achilles tendon pathology, chronic tendinopathic tendons, insertional Achilles pathologic entities, insertional calcific Achilles tendinosis, mid-tendinous Achilles tendinopathy, tenosynovitis of the tendo Achilles and tuberculous tenosynovitis of the Achilles tendon, Haglund's triad

**Fig. 2:**
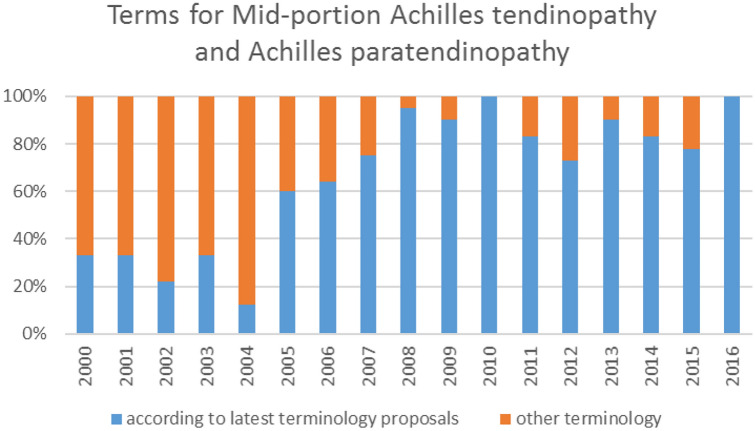
The distribution of terms used or mid-portion Achilles tendinopathy and Achilles paratendinopathy in percentages of published articles (Y-axis) over the years (X-axis)

**Fig. 3:**
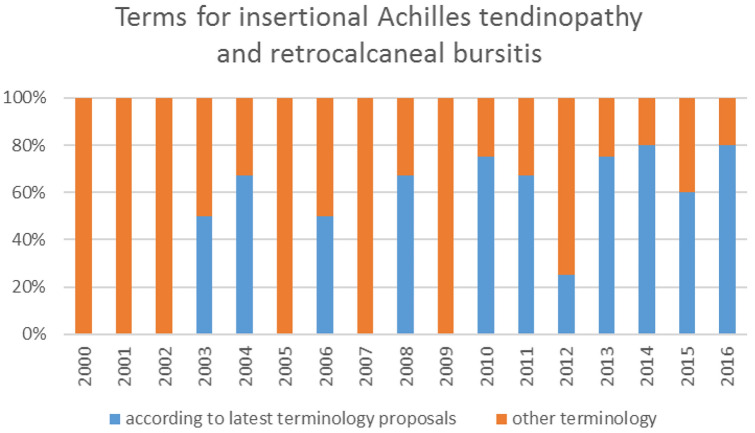
The distribution of terms used for insertional Achilles tendinopathy and retrocalcaneal bursitis in percentages of published articles (Y-axis) over the years (X-axis)

Figure 2 shows the distribution of terms used for mid-portion Achilles tendinopathy and Achilles paratendinopathy in percentages from 2000 to 2016. In 2000, 33% uses terminology according to the latest proposals and in 2016, 100%. The distribution of terms used for insertional Achilles tendinopathy and retrocalcaneal bursitis is shown in Fig. 3, in 2000, 0% uses terminology according to the latest proposals and in 2016, 80%.

## Discussion

The main findings of this study were that terminology for Achilles tendon-related disorders according to the latest proposals based on anatomic location, symptoms, clinical findings and histopathology is being used by the majority of orthopedic surgeons in daily practice and is increasingly being used in the literature. However, the indistinct Haglund eponyms are still frequently used in Achilles tendon-related terminology.

The wide variety in terminology for Achilles tendon-related disorders is confusing. The term that represents the entity must be neutral yet descriptive, uniform and clear. Therefore, descriptive terms are preferable to eponymous terms [21]. Terminology which includes the combination of anatomic location, symptoms and clinical findings and pathological changes for each entity has, therefore, been advocated.

Symptoms around the Achilles tendon often have a similar presentation and it is, therefore, important to define the pathology or the combination of pathologies. For example, lack of distinction between entities, such as insertional tendinopathy and chronic retrocalcaneal bursitis is crucial to determine further treatment and it impedes the process for researchers to perform an all-encompassing systematic review [3, 27].

In five out of six cases in the survey, the majority of orthopedic surgeons gave a diagnosis according to the terminology based on anatomic location, symptoms, clinical findings and histopathology. The exception is the fifth case, where the majority choose Haglund’s disease instead of retrocalcaneal bursitis. A possible reason for this is the ingrained use of the eponym Haglund. There are approximately 20,000 medical eponymous terms in use today and the literature shows that using eponymous terms is an inaccurate and unreliable method of communication [4, 5, 21]. Somford et al. questioned 244 orthopedic surgeons worldwide on common eponymous terms and reported a low agreement on use of eponymous terms (kappa 0.11; proportion of agreement, 68%). Nevertheless, eponymous terms are often used in clinical setting and are passed onto the residents and students [11, 15, 22, 26]. Also, eponymous terms used in the published articles are often inconsistent and do not match their original definition [20, 23].

Terminology in which Haglund eponyms such as Haglund’s deformity, Haglund’s syndrome and Haglund’s disease are all dissimilar entities should be avoided, because there is a large variation in the presumed meaning of these eponymous terms [21]. Haglund’s syndrome was first defined as a common cause of posterior heel pain, characterized clinically by a painful soft tissue swelling at the level of the Achilles tendon insertion [13]. Haglund’s deformity was first described as a tender swelling in the region of the Achilles tendon with visible prominence of the postero-lateral aspect of the calcaneus [25]. Haglund’s disease, however, refers to osteochondrosis of the accessory navicular bone [6, 19].

In systematic reviews, many eponymous diagnosis have to be converted to anatomical diagnostic groupings and at all studies are excluded based on aberrant or uninterpretable definitions of an eponym or pathology, which can lead to different research results which are often leading for the best scientific-based treatment in clinical practice [2, 9, 27].

The survey was sent to members of the Ankleplatform Study Group, which caused selection bias. Even though orthopaedic surgeons from over the whole world responded, these were specifically experienced in the field of foot and ankle pathology what could have led to an overestimation of the terms used compared by orthopaedic surgeons in general. Also, the presumed definitions of the terms used for Achilles tendon-related disorders were not assessed which could have provided insight into the misuse of terms. In the literature study, we included a selection of eight foot and ankle journals, which caused selection bias.

Uniform terminology provides the ability to communicate with an universal language in daily practice amongst clinicians and researchers and will lead to the best available scientific-based treatment in clinical practice.

## Conclusion

The revised terminology for Achilles tendon-related disorders is used by the majority of orthopedic surgeons and is increasingly used in the literature. However, the indistinct Haglund eponyms are still frequently used in Achilles tendon-related terminology.

## Supplementary Information

Below is the link to the electronic supplementary material.

Supplementary file 1 (DOCX 402 kb)

Supplementary file 2 (DOCX 12 kb)
