# Supplementary material for: Increasing consensus on terminology of Achilles tendon-related disorders
Source: Knee Surg Sports Traumatol Arthrosc. 2021 May 15;29(8):2528–34. doi: 10.1007/s00167-021-06566-z (PMC8298365; doi:10.1007/s00167-021-06566-z)
Supplement: Supplementary file 1 — (DOCX 402 kb) [file 167_2021_6566_MOESM1_ESM.docx]

**Appendix I.**

**Case 1**


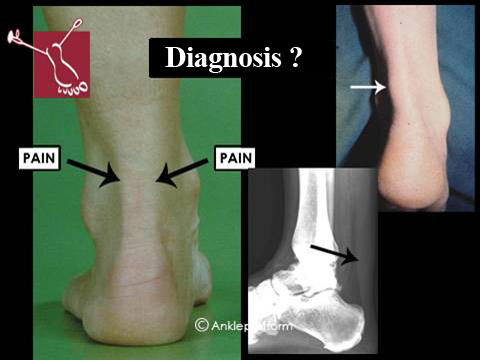


The photo on the left shows the location of the pain in a normal ankle. The photos on the right show the pathology.

- 40-year-old male.

- Pain over his right Achilles tendon, which impairs his activity.

- Painful localized fusiform swelling of the Achilles tendon located 6 cm proximal to the insertion onto the calcaneus.

- X-ray shows deviation of soft tissue contours.

- Ultrasound showed a larger tendon than normal (in cross-sectional area and antero-posterior diameter). There were hypoechoic areas within the Achilles tendon and increased tendon vascularity (mainly in the ventral peritendinous area).

What is your diagnosis? **Mid-portion Achilles tendinopathy**

**Case 2**


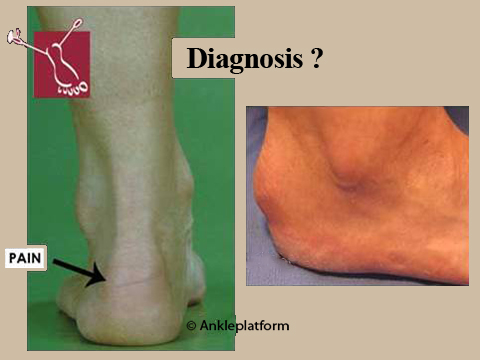


The photo on the left shows the location of the pain in a normal ankle. The photos on the right show the pathology.

- 30-year-old female.

- Posterolateral heel pain on the right side (worsens with shoes with rigid posterior portion).

- Visible, painful and solid swelling with discoloration of the skin located at the posterolateral calcaneus.

- X-ray shows no abnormality.

- Ultrasound showed fluid between the skin and the Achilles tendon.

What is your diagnosis? **Superficial calcaneal bursitis**

**Case 3**


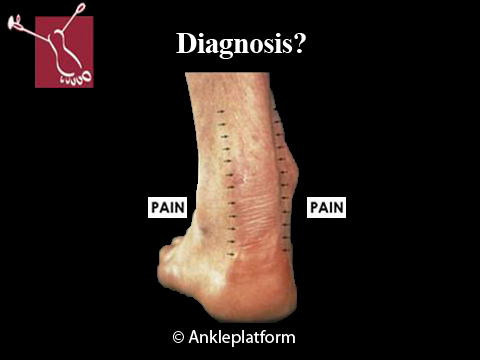


- 32-year-old male.

- Since 4 days edema and hyperemia around the midportion Achilles tendon on the left side.

- Redness and swelling of the skin over the full length of the Achilles tendon. .

- On palpation there are crepitations.

- X-ray shows no abnormalities.

- Ultrasound showed a normal Achilles tendon with a circumferential hypoechogenic halo.

What is your diagnosis? **Acute Achilles paratendinopathy**

**Case 4**


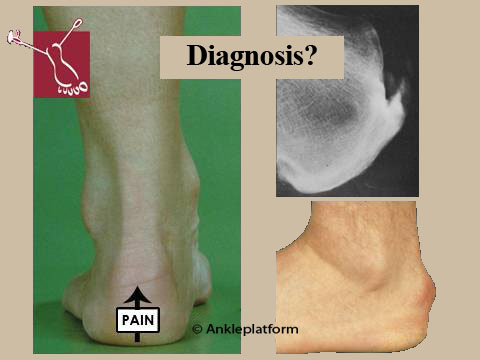


The photo on the left shows the location of the pain in a normal ankle. The photos on the right show the pathology.

- 60-year-old male.

- Stiffness and pain on the posterior aspect of the calcaneus on the left foot.

- Painful Achilles tendon insertion at the mid-portion of the posterior aspect of the calcaneus with visible swelling

- X-ray shows calcaneal bone spur at the insertion of the Achilles tendon.

What is your diagnosis? **Insertional Achilles tendinopathy**

**Case 5**


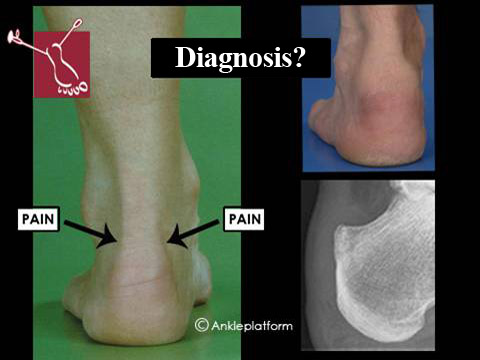


The photo on the left shows the location of the pain in a normal ankle. The photos on the right show the pathology.

- 35-year-old female patient.

- Painful swelling superior to the calcaneus of the left foot.

- Painful soft tissue swelling, medial and lateral to the Achilles tendon at the level of the posterosuperior calcaneus.

- X-ray shows bony prominence of the posterosuperior calcaneus.

- Ultrasound showed fluid in the retrocalcaneal area (hyperechoic).

What is your diagnosis? **Retrocalcaneal bursitis**

**Case 6**


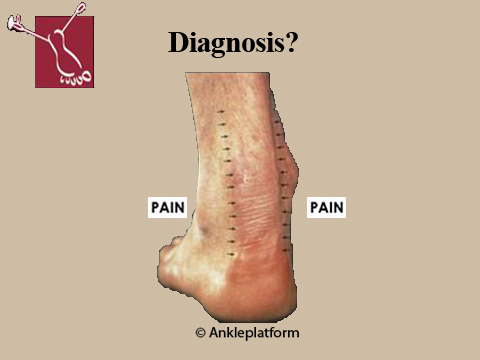


- 39-year-old male.

- Since one year exercise-induced pain around the midportion Achilles tendon of the left foot.

- Minimal swelling and some crepitations around the midportion Achilles tendon.

- X-ray shows no abnormalities.

- Ultrasound shows thickened hypoechoic paratenon and the echo-Doppler shows increased tendon vascularity (mainly in ventral peritendinous area).

What is your diagnosis? **Chronic Achilles paratendinopathy**
