# Supplementary material for: Increasing consensus on terminology of Achilles tendon-related disorders
Source: Knee Surg Sports Traumatol Arthrosc. 2021 May 15;29(8):2528–34. doi: 10.1007/s00167-021-06566-z (PMC8298365; doi:10.1007/s00167-021-06566-z)
Supplement: Supplementary file 2 — (DOCX 12 kb) [file 167_2021_6566_MOESM2_ESM.docx]

**Appendix II**

*Search strategy*

A systematic literature search was performed in Medline, Embase (Classic). A search with the following keywords was performed: *“Achilles tendinitis OR Achilles tendinopathy OR Achilles tendinosis OR Achilles tendon bursitis OR Achillodynia OR Achillotendinitis ossificans OR Acute Achilles paratendinopathy OR Bursitis Achillea OR Cellulite peritendineuse Achilles tendon OR Chronic Achilles paratendinopathy OR Haglund’s deformity OR Haglund’s disease OR Haglund's exostosis OR pump-bump OR calcaneus altus OR high prow heels OR knobbly heels OR cucumber heel OR Haglund’s syndrome OR Insertional Achilles tendinopathy OR Mid-portion Achilles tendinopathy OR Paratendinitis Achilles OR Peritendinitis Achilles OR Retrocalcaneal bursitis OR Superficial Calcaneal bursitis OR Tendinitis Achillea traumatica OR Tenosynovitis Achilles OR non-insertional Achilles tendinopathy OR noninsertional Achilles OR Midportion Achilles tendinopathy”*.
